# A simulation framework for bio-inspired sonar sensing with Unmanned Aerial Vehicles

**DOI:** 10.1371/journal.pone.0241443

**Published:** 2020-11-03

**Authors:** M. Hassan Tanveer, Xiaowei Wu, Antony Thomas, Chen Ming, Rolf Müller, Pratap Tokekar, Hongxiao Zhu

**Affiliations:** 1 Department of Statistics, Virginia Tech, Blacksburg, Virginia, United States of America; 2 Department of Robotics & Mechatronics Engineering, Kennesaw State University, Marietta, Georgia, United States of America; 3 DIBRIS, University of Genova, Genova, Italy; 4 Department of Neuroscience, Brown University, Providence, Rhode Island, United States of America; 5 Department of Mechanical Engineering, Virginia Tech, Blacksburg, Virginia, United States of America; 6 Department of Computer Science, University of Maryland, College Park, Maryland, United States of America; Oak Ridge National Laboratory, UNITED STATES

## Abstract

We introduce a unified simulation framework that generates natural sensing environments and produces biosonar echoes under various sensing scenarios. This framework produces rich sensory data with environmental information completely known, thus can be used for the training of robotic algorithms for biosonar-based Unmanned Aerial Vehicles. The simulated environment consists of random trees with full geometry of the tree foliage. To simulate a single tree, we adopt the Lindenmayer system to generate the initial branching pattern and integrate that with the available measurements of the 3D computer-aided design object files to create natural-looking branches, sub-branches, and leaves. A forest is formed by simulating trees at random locations generated by using an inhomogeneous Poisson process. While our simulated environments can be generally used for testing other sensors and training robotic algorithms, in this study we focus on testing bat-inspired Unmanned Aerial Vehicles that recreate bat’s flying behavior through biosonar sensors. To this end, we also introduce an foliage echo simulator that produces biosonar echoes while mimicking bat’s biosonar system. We demonstrate the application of the proposed simulation framework by generating real-world scenarios with multiple trees and computing the resulting impulse responses under static or dynamic motions of an Unmanned Aerial Vehicle.

## Introduction

Many environments, such as dense vegetation and narrow caves, are not easily accessible by human beings. Unmanned Aerial Vehicles (UAVs) provide cost-effective alternatives to human beings for a large variety of tasks in such environments, including search, rescue, surveillance, and land inspection. In recent years, impressive progress has been made in UAVs, leading to revolutions in the aerodynamic structure, mechanical transmission, actuator, computer control, etc. Despite these advances, existing technology in UAVs is still limited as most systems can only operate in clear, open space [1] or in fields with sparsely distributed tree obstacles [2], and most existing approaches for localization and path planning fail in the presence of large number of obstacles. Moreover, sensors used in these systems are often bulky which hinders efficient navigation [3]. It is highly desirable to build safe and efficient UAV systems that do not fail under complex, real-world conditions.

Among many directions in technological innovation, bio-inspired technology provides a promising solution that may break the performance boundary in UAVs. Mammals, insects, and other organisms often exhibit advanced capabilities and features that would be desirable for UAVs. They can rapidly pick out salient features buried in large amounts of data, and adapt themselves to the dynamics of their surrounding environments. Adopting prototypes that emulate the characteristics and functions found in living creatures may enable robots to maneuver more efficiently without the aid of approaches such as simultaneous mapping and localization (SLAM), Global Positioning System (GPS) or inertial units. In recent years, bio-inspired approaches have already given rise to robots that operate in water [4], air [5], on land [6] and, in some cases, transiting in various media. For UAVs in particular, Microbot has been developed by [7] which achieves independent fly by imitating the morphological properties of versatile bat wings. In 2011, AeroVironment successfully developed the “Hummingbird” by mimicking hummingbirds [8]. Besides these examples, there are several other conventional designs developed, including Robird [9], DelFly [10], and Bat Bot [11].

In this study, we consider using the echolocation system of bats as a biological model for the study of highly parsimonious biosonar sensors for UAVs. Echolocating bats perform parsimonious sensing in complex natural environments with small, low cost transducers. They employ miniature sonar systems with a few transducers—a nose (or mouth) and two ears, yet achieve much better navigation performance than engineered systems. Specifically, an echolocating bat emits brief ultrasonic pulses through mouth or nostrils, and use the returning echoes to navigate [12, 13]. Due to their outstanding navigation ability, bats have received constant attention in the research of bio-inspired radar and sonar [14, 15]. Robotic systems that mimic bat’s biosonar have been designed to accomplish various tasks including localization and identification of objects [16–19], tracking [20], obstacle-avoidance [21, 22], safe landing [23], and convoy control [24].

Despite the progress, existing studies primarily focus on designing biosonar-based UAVs for specific tasks such as landmark identification and obstacle avoidance, and testing of these systems is often performed in environments with spatially isolated objects. For example, authors in [19] focused on localizing reflectors with the shapes of a ball, a block, and a crumpled paper ball; authors in [21] evaluated the performance of their system on obstacle avoidance by using randomly distributed plastic poles in a chamber. Moreover, due to the limitation of experiments, training and testing can only be performed under limited experimental conditions such as human-designed lab environments or pre-selected natural experimental sites. For example, in [20] and [18], artificial trees were used to test the performance of the proposed robot navigation system on landmark tracking; in [21], a chamber was used to design a sensing environment; and in [25], two greenhouses were selected as the experimental sites to test a terrestrial robot. If there is a simulation tool that can generate random sensing environments and simulate biosonar sensors, one can simulate more complicated sensing scenarios including those that are hard to be created under experimental setups. Biosonar signals can be generated in real time and combined with the known environmental information for the training of online learning and control algorithms. For example, when simulating a bat chasing a prey in a dense forest, biosonar echoes can be collected over time in an adaptive manner and used to estimate edges of the accessible routes, update status of the prey, and control the UAV. The trained algorithms can be used on biosonar-based UAVs that recreate bats’ abilities.

With the availability of modern computing infrastructure and the development of big data analytics, we believe that it is now possible to build virtual simulation platforms for the development of biosonar-based UAVs and other smart sensing systems. In contrast with experimental approach, noted advantages of virtual simulation include its low-cost, flexibility, and repeatability. For example, the same sensing scenario can be repeated many times under similar random environmental setups, and different sensing tasks can be performed under the same sensing environment. To create such a virtual simulation platform, we propose a unified simulation framework that generates natural sensing environments and produces biosonar echoes under various sensing scenarios. This framework produces rich sensory data with environmental information completely known, thus can be used for the training of robotic algorithms for biosonar-based UAVs.

Our proposed simulation framework consists of two types of simulators, one for the simulation of sensing environment which produces random trees with necessary geometry (leaf locations, size, and orientations etc.), the other for the simulation of foliage echoes. While numerous tree models are available [26–31], simulating the full geometry of a natural-looking tree remains a challenging task. Tree models in available software often rely on plugins (e.g., Blender tree plugins), which do not provide detailed geometric information. Empirical tree models that are based on real tree templates are sometimes useful, but they are often hard to be randomized. Additionally, due to the large variety of tree species, simulating tree structures of a large family of tree types is challenging. To address these challenges, we propose an efficient approach by integrating Lindenmayer systems (L-systems) with 3D Computer-Aided Design (CAD) object files. The L-system [32] is a graphical model commonly used to define the branching patterns in trees and other organic forms [33]. It defines the branching pattern through recursively applying certain production rules on a string of symbols. While being an ideal mathematical model for branching patterns, the L-system often contains over-simplified assumptions on the geometry of branches, sub-branches, and leaves. We therefore further improve the L-system by including geometric structures available in 3D CAD developed object files. A 3D CAD file represents objects by using triangular faces, and each triangular face comprises a normal direction and the coordinates of the three vertices that form the triangle. They are created based on real tree templates, thus contain more detailed geometric parameters (such as natural branch curvatures and leaf orientations). Moreover, since there is a large amount of 3D CAD object files available, simulating different tree species becomes straightforward. For example, an excellent source of maple tree files can be found via the link https://www.turbosquid.com/3d-model/maple-tree. We add randomization to the placements of branches, sub branches, and leaves to create random trees with natural looks. Finally, to simulate a forest, we adopt an inhomogeneous Poisson process (IPP) [34] to generate random locations of trees.

While our simulated environment can be used as a general platform for studying the sensing mechanism of different sensing systems, our focus is on the training of bat-inspired UAVs that can recreate bat’s flying behavior (e.g., obstacle avoidance, path planning) in dense vegetation. To this end, we also introduce an foliage echo simulator that can produce simulated echoes by mimicking bat’s biosonar. It is constructed following acoustic laws of sound emission, propagation, and reflection. It takes into account both the biosonar beampattern and the geometric properties of the reflectors. With the simulated environment and the foliage echo simulator, we are able to simulate different sensing scenarios and compute the corresponding impulse responses along the flying trajectories of a UAV. We will demonstrate the application of the proposed simulation framework by simulating real-world scenarios with multiple trees and computing the resulting impulse responses under static or dynamic motions of a UAV.

## Materials and methods

### The simulation of a natural-looking tree

**Fig 1:**
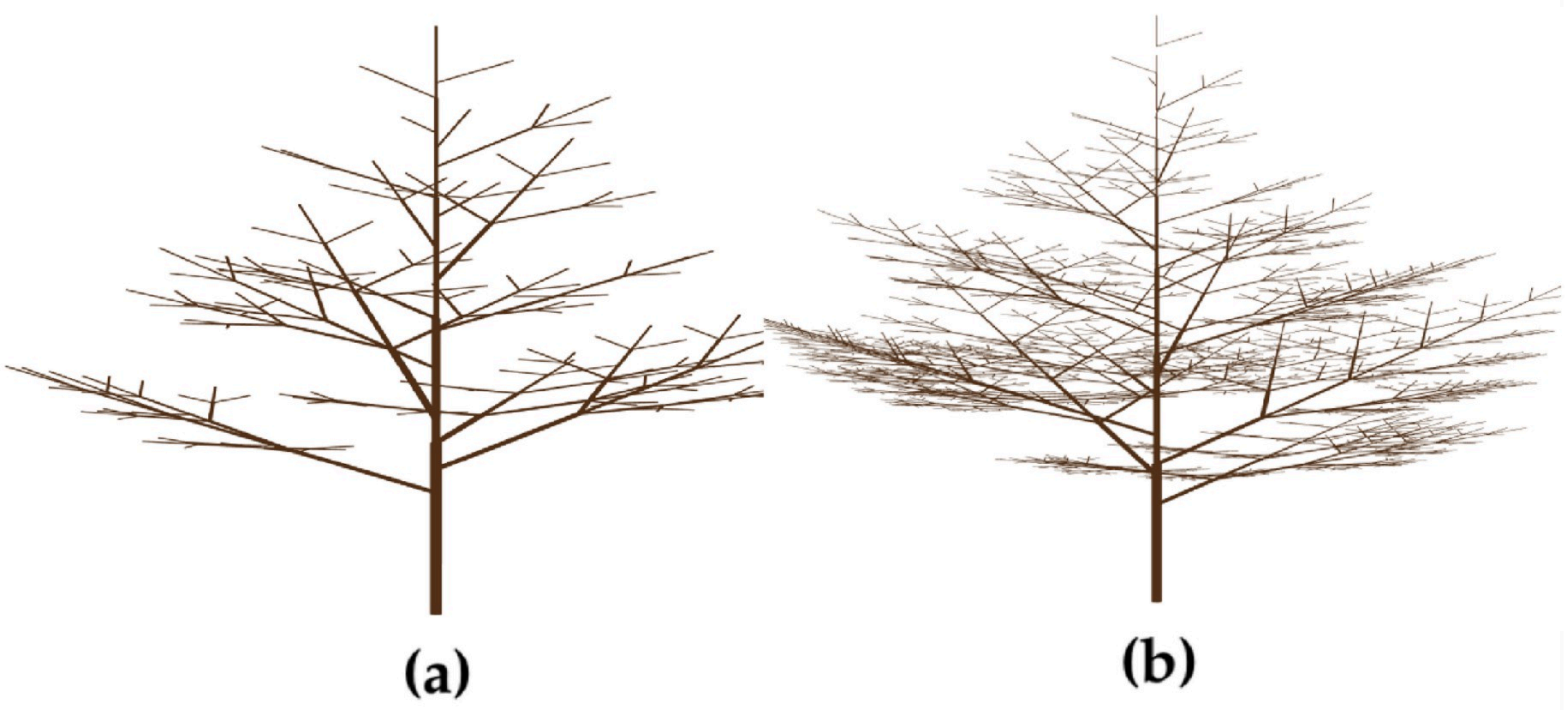
The branching structures of trees generated by a L-system. (a) The structure with four iterations. (b) The structure with six iterations.

We simulate the first level branching structure of each individual tree by the L-system. We denote an L-system by a tuple **G** = (*A*, *ω*, Ω, Σ, *P*), where *A* is a finite set of symbols or variables; *ω*, also called the axiom, is a string of symbols from *A* that defines the initial state of the system. The alphabet Ω = {(,), +, [} is a set of special symbols that define specific instructions for branching, and Σ = *A*∪Ω. Finally, *P*: *ω* → Σ^⋆^ defines production rules that describe the transformation of the axiom variables into strings. Here, Σ^⋆^ denotes the set of strings over the alphabet Σ. Different production rules can be defined for different branching patterns. For example, let *A* = {*g*, *d*} denote the variable set where *g* means a starting/end branch of the tree (a leaf is simplified as an end branch) and *d* means a trunk/branch, and let {*ω*} = {*g*} denote the axiom. We may define a production rule *P*: *g* → *d*(*g*)[*g*) + *g*), which means that starting from the axiom *g*, the first iteration gives the branching pattern *d*(*g*)[*g*) + *g*), and the second iteration gives an overall pattern *d*(*d*(*g*)[*g*) + *g*)[*d*(*g*)[*g*) + *g*) + *d*(*g*)[*g*) + *g*). Note that to obtain the pattern in the second iteration, the variable *g* in the first branching pattern is replaced by the production rule, *g* → *d*(*g*)[*g*) + *g*). The branching pattern created by using four and six iterations are shown in Fig 1(a) and 1(b). Here, the symbol “(” means to create a branch starting point, “)” means to create a branch end point, “+” creates a new branch at the symmetric position of the previous branch, and “[” increases the height of the trunk and start a new branch. When creating a symmetric branch, the new starting point was raised by a random height to avoid perfect symmetric structure. The branch is assigned an initial length and radius. These parameters change according to certain ratios as the tree grows. We randomize each contraction ratio by adding a random noise to a fixed ratio. Angles between the branches are also defined in a similar fashion.

**Fig 2:**
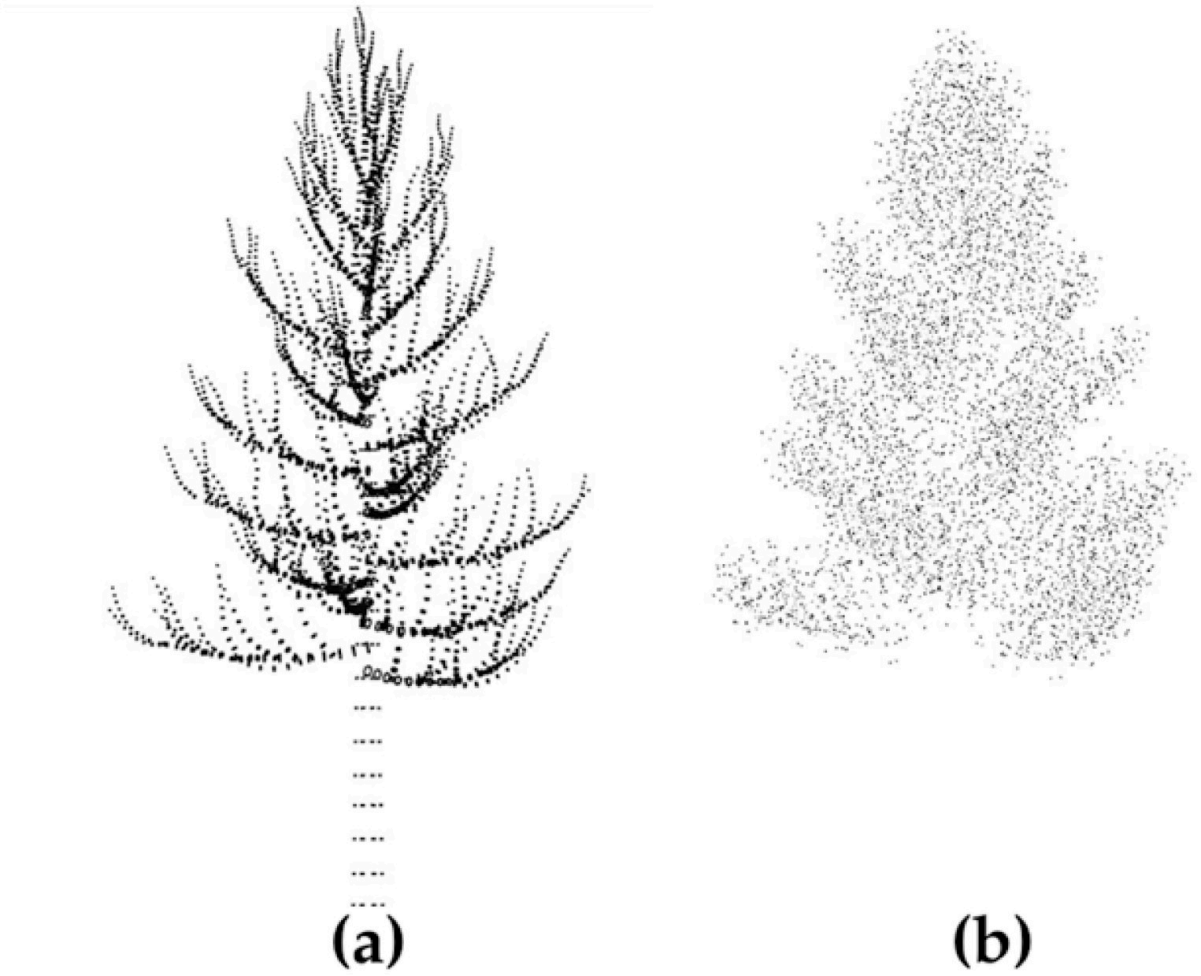
The 3D model of a maple tree visualized in a CAD viewer. (a) The branch structures, with vertices of surface meshes plotted as a point cloud. (b) The tree leaves plotted as triangular meshes.

Despite their effectiveness, most L-system models rely on a few parameters to control the branching structure and lengths/thickness/angles of branches. Although probability distributions can be introduced to randomize these parameters, they are often not enough to characterize all features of a particular tree species. For this reason, we choose to adopt an L-system to generate the first level branching locations at the trunk, and generate the branches and sub-branches by modifying reference trees from CAD developed object files. CAD is a way of creating models of objects using computers. A 3D model designed using CAD contains all details required to make a 3D print of the model. One type of 3D CAD files that is commonly used in computer games and movies is the STereoLithography (STL) file, which contains polygonal meshes defined by vertices and normal vectors that represent the surface features of the 3D object. As the simulation of foliage echo requires information such as the location, size, and orientation of leaves, we choose to use STL files to represent trees. In Fig 2, we demonstrate the visualization of a tree in a CAD viewer.

After the trunk and branch positions are generated from the L-system, CAD files that contain information about branches (including sub-branches and leaf parameters) are extracted. The branches are translated and rotated so that the root positions match those generated by the L-system. For example, consider the case that the CAD object file has *n* branches and L-system generates *N* branches. If *n* = *N*, we simply make a one-to-one match with slight randomization of the parameters by adding a random noise to all branch/leaf parameters. If *n* ≠ *N*, we construct all branches by randomizing a randomly selected template branch via rotation, translation and scaling. In case of different species, we design different L-systems for each species and take advantage of CAD data from the corresponding species when simulating trees.

### Simulation of a forest

The model for single-tree simulation can be applied repeatedly to produce multiple trees of different species. This can be done through parallel computing to save computation time. To form a forest, we need to model the distribution of trees in a field. We achieve this through inhomogeneous Poisson process, abbreviated as IPP, because of its flexibility and computational convenience. An IPP models the distribution of random points in space or random “events” in a time interval. It can be used to model a multitude of spatial and temporal phenomena, such as cars passing through a junction or the timing/place of animal sightings. By sampling from IPP, we can determine the number of trees as well as the positions of these trees in a 2-D field. Specifically, let $D\in R^{2}$ denote the field on which the group of trees will be simulated. The random locations (i.e., (*x*, *y*) coordinates) of the trees will be denoted by *S* = {*s*_*i*_, *i* = 1, …, *n*}. We assume that *S* follows an IPP with intensity function *λ*(*s*):*D* → *R*^+^, where *λ*(*s*) is a parameter that control the tree density on *D*. Small values of *λ*(*s*) indicate sparse regions whereas high values indicate dense regions. Given the region *D* and the intensity *λ*(*s*), the number of trees, *n*, follows a Poisson distribution with mean ∫_*D*_ *λ*(*s*)*ds*. To simulate *S* given *n*, we adopt a thinning approach; details can be found in [35].

**Fig 3:**
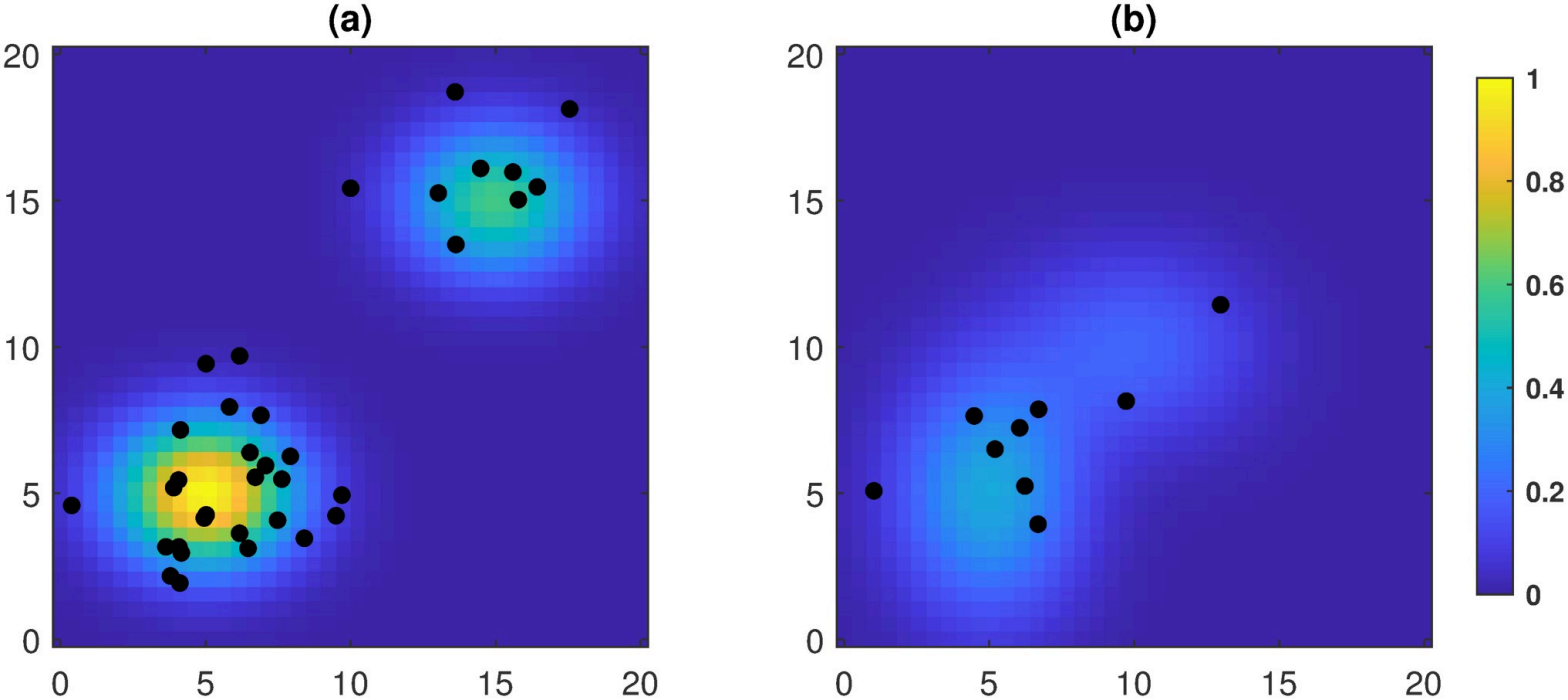
Samples from IPPs with a mixture of squared exponential intensity function (*p* = 2). (a) A sample corresponds to *C*_1_ = 1.0, *C*_2_ = 0.6, (*a*_1_, *b*_1_) = (5, 5), (*a*_2_, *b*_2_) = (15, 15), and (*h*_1_, *l*_1_) = (*h*_2_, *l*_2_) = (3, 2). (b) A sample corresponds to *C*_1_ = 0.4, *C*_2_ = 0.2, (*a*_1_, *b*_1_) = (5, 5), (*a*_2_, *b*_2_) = (10, 10), (*h*_1_, *l*_1_) = (3, 4), and $\left(h_{2},l_{2}\right)=\left(4,\sqrt{10}\right)$.

The intensity function *λ*(*s*), *s* ∈ *D* is an input of the forest simulator. Its format should be specified by the user. For example, one way to specify *λ*(*s*) is to use a mixture of squared exponential (Gaussian) kernel functions, i.e., $\lambda \left(s\right)=\sum_{i=1}^{p}C_{i}\exp\left\{-{\left(s_{x}-a_{i}\right)}^{2}/h_{i}^{2}-{\left(s_{y}-b_{i}\right)}^{2}/l_{i}^{2}\right\}$, where *p* is the number of mixing component, (*s*_*x*_, *s*_*y*_) denotes the (*x*, *y*) coordinates of *s*, (*a*_*i*_, *b*_*i*_) is the center of the *i*th mixing component, (*h*_*i*_, *l*_*i*_) are positive numbers that control the standard deviation of each mixing component, and *C*_*i*_ is a scaling parameter. The values of (*a*_*i*_, *b*_*i*_), (*h*_*i*_, *l*_*i*_) can be determined by visualizing the location and the shape of each mixing component, and *C*_*i*_ can be determined by controlling the expected total number of trees in the field and the expected proportion of trees in each mixing component. In Fig 3, we demonstrate samples from IPPs with mixed squared exponential intensity functions (*p* = 2), where the two plots (a) and (b) correspond to *λ*(*s*) with different parameter setups.

In some cases, if there is a real experimental field that can serve as a template of the simulation and the location of each tree is known, the value of *λ*(*s*) can be estimated based on the spatial data collected from the experimental field. Various estimation methods, such as those developed by [36, 37] and [38, 39], can be used for estimating *λ*(*s*). If there are more than one plant species, the intensity estimation and simulation should be performed separately for each species. In this case, a merging step is required to pool all species into one community, and extra restrictions will be applied to ensure, for example, two trees of different species are not too close to each other.

Comparing with existing methods such as models that assume homogeneous tree distribution [40–42] and models that are based on modeling local interactions between individual plants [43–45], the IPP approach described above offers several unique features and advantages. First, as a general stochastic model, it generates flexible inhomogeneous spatial distributions for plants of different species. Second, the IPP sampling is automatic, computationally efficient, and does not require users to manually interfere with the sampling procedure. Third, if desired, the input—the intensity function—can be estimated based on a real experimental site, making it possible to mimic a real experimental site in simulation. Finally, the sampling procedure can be repeated as many times as needed, producing plant distributions with similar spatial characteristics.

### Generating foliage echoes

Foliage echoes received by biosonar can depend on many factors such as the sonar beampattern, the configuration of the vegetation, etc. We adopt a computational model to simulate foliage echoes from a broad range of species following [46] and [47]. Our model mimics the echolocation process by sending a signal to the reflectors in space and calculating the reflected signal of each reflector. The reflection rule takes into account parameters such as the distance to the sonar and the orientation and size of each reflector. The reflected signals will finally be added up at the position of sonar, and the result of superposition is one echo.

In order to adopt acoustic laws, several simplifications were made. First, leaves are approximated by circular discs. The midpoint of each disc is chosen to be the midpoint of the triangular mesh that constitutes a leaf in the CAD file, and the radius is generated from a normal distribution. The scattering of each leaf is calculated from spheroidal wave functions [48, 49] and later approximated with a cosine function. The sonar beam is modeled by a 2D Gaussian function following the study of [50] about bat’s sonar beams. Furthermore, we neglect shading effects between leaves. The effect of these simplification to the simulated echoes were studied by [47] by comparing the summary statistics of simulation echoes with those of the experimental echoes. Additionally, the sonar was assumed to be monostatic, i.e., the emitter and the receiver were in the same position. This assumption makes sense when the size of sonar is as small as a bat’s head, in which case the distance between the transmitter and the receiver is small (e.g., a few centimeters) compared to the distance to the target.

More specifically, the simulated foliage echoes are generated as time-domain signals. Let *Y* = {*y*_1_, …, *y*_*n*_} denote a time-domain echo signal to be simulated. Let $Y^{*}=\left\{y_{1}^{*},\ldots ,y_{n^{{}^{\prime }}}^{*}\right\}$ denote the Fourier transform of *Y* in the frequency domain. To obtain *Y*, we first compute Y* and apply inverse fast Fourier transform. We assume that $y_{k}^{*}$ is nonzero in the frequency range between 60 and 80 kHz, which matches the terminal frequency modulated (tFM) portion at the end of echolocating signals of greater horseshoe bats (*Rhinolophus ferrumequinum*) [51]. This tFM part has been shown effective for the precise localization of a target. Let $y_{k}^{*}$ denote the Fourier component corresponding to the frequency *f*_*k*_. According to acoustic laws of sound reflection [49], each Fourier component $y_{k}^{*}$ is the superposition of all the reflected echoes from the reflecting facets within the main lobe of the sonar. Each $y_{k}^{*}$ takes the form

$$\begin{array}{c} y_{k}^{*}=\sum_{i=1}^{m}A_{ki}\cos\left(\phi_{ki}\right)+j\sum_{i=1}^{m}A_{ki}\sin\left(\phi_{ki}\right), \end{array}$$

where *j* is the imaginary unit, *m* denotes the number of reflecting facets within the main lobe of the sonar, *A*_*ki*_ is the amplitude at frequency *f*_*k*_ corresponding to the *i*-th facet, and *ϕ*_*ki*_ is a phase delay parameter at *f*_*k*_ for the *i*-th facet. The amplitude *A*_*ki*_ is computed by

$$\begin{array}{c} A_{ki}=S\left(az_{i},el_{i},A_{0}\right)L_{i}\left(\beta_{i},a_{i},f_{k}\right)\frac{\lambda_{k}}{2\pi r_{i}^{2}}, \end{array}$$

where *S*(*az*_*i*_, *el*_*i*_, *A*_0_) represents the sonar beampattern; *az*_*i*_ and *el*_*i*_ denote the azimuth and elevation angles of *i*-th facet relative to the sonar; *r*_*i*_ is the distance between the sonar and the *i*-th reflecting facet; *λ*_*k*_ is the wavelength of the emitted sound wave corresponding to the frequency *f*_*k*_; and *L*_*i*_(*β*_*i*_, *a*_*i*_, *f*_*k*_) denotes the beampattern of the reflecting facet with *β*_*i*_ and *a*_*i*_ denoting the incident angle and radius of the *i*-th reflecting facet respectively. The sonar beampatern *S*(*az*_*i*_, *el*_*i*_, *A*_0_) is calculated by using a Gaussian function to approximate the mainlobe of an actual bat’s beampattern. The Gaussian function takes the form

$$\begin{array}{c} S\left(az_{i},el_{i},A_{0}\right)\approx A_{0}\exp\{-[\frac{{\left(az_{i}-az_{0}\right)}^{2}}{2\sigma_{x}^{2}}+\frac{{\left(el_{i}-el_{0}\right)}^{2}}{2\sigma_{y}^{2}}]\}, \end{array}$$

where the parameter *A*_0_ denotes the maximum amplitude of the beampattern along the beam direction, *az*_0_ and *el*_0_ are the azimuth and elevation of the beam direction, and *σ*_*x*_ and *σ*_*y*_ are the spreads of the Gaussian blob which are determined by the beamwidth estimated from empirical data of horseshoe bats.

Calculation of the leaf beampattern *L*_*i*_(*β*_*i*_, *a*_*i*_, *f*_*k*_) requires numerically evaluating the scattered field of a single disc, which is computationally intensive. In order to improve computation efficiency, we approximate *L*_*i*_(*β*_*i*_, *a*_*i*_, *f*_*k*_) by using a cosine function of the form

$$\begin{array}{c} L_{i}\left(\beta_{i},a_{i},f_{k}\right)\approx P_{1}(c(f_{k},a_{i}))\cdot \cos(P_{2}(c(f_{k},a_{i}))\cdot \beta_{i}), \end{array}$$

where *c*(*f*_*k*_, *a*_*i*_) = 2*πa*_*i*_ *f*_*k*_/*v* with *v* being the speed of sound, and *P*_1_ and *P*_2_ are polynomials of *c*. The forms of *P*_1_ and *P*_2_ are approximated by fitting nonlinear regressions based on data obtained from numerical evaluations. In particular, *P*_1_(*c*) = 0.5003*c*^2^ + 0.6867 and *P*_2_(*c*) = 0.3999*c*^−0.9065^ + 0.9979. More details about the approximation can be found in [46].

**Fig 4:**
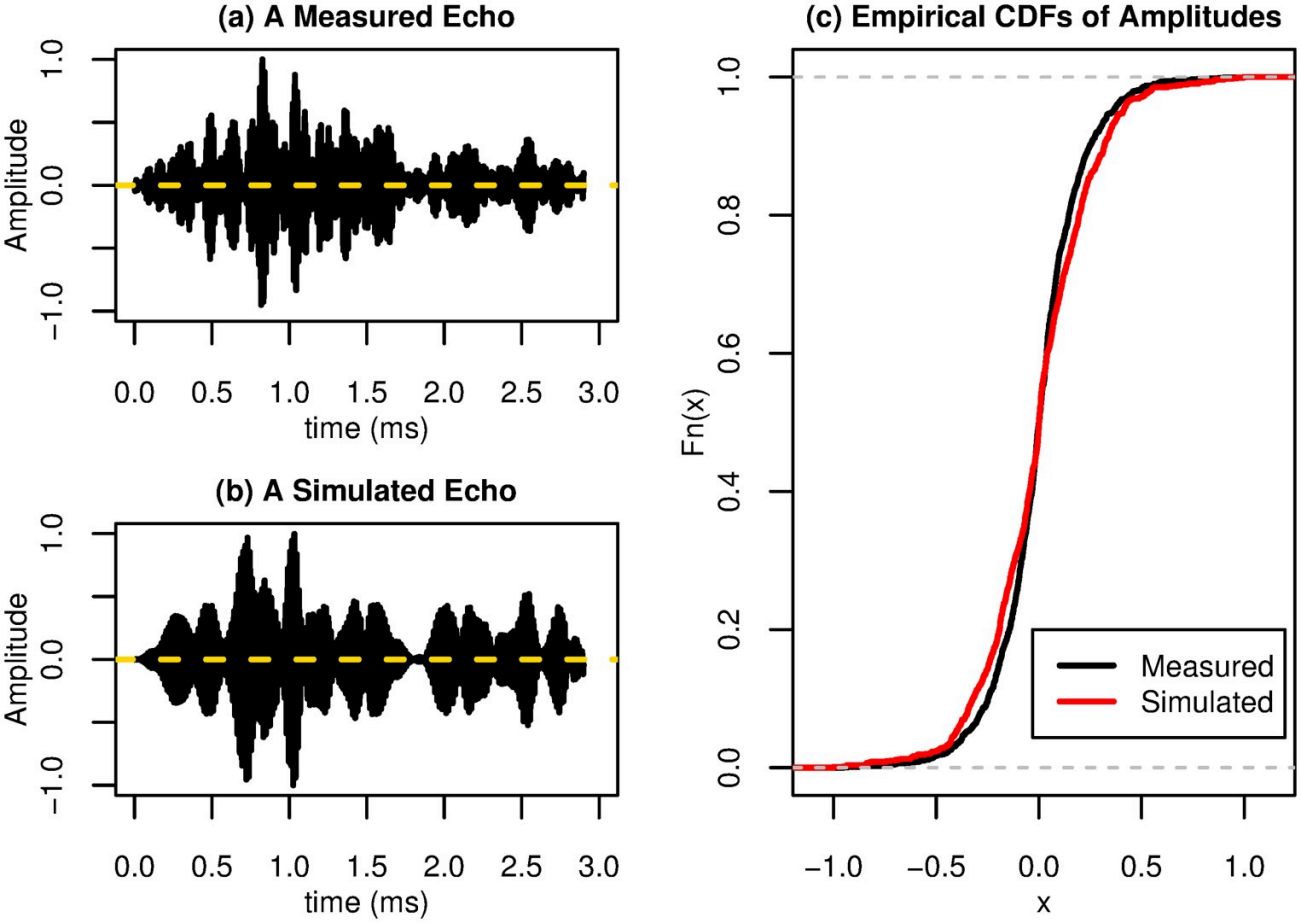
A comparison between simulated and measured echoes for a maple tree. (a) An echo signal measured from a maple tree. (b) An simulated echo signal obtained by matching the simulation setup with the experimental setup. (c) Plots of the empirical CDFs of the signal amplitudes.

In order to validate that simulated echoes resemble echoes collected with real bio-sonar sensors, a preliminary validation study has been performed by our coauthors [46]. In the study, echoes from two different tree species were measured by using a static biomimetic sonar head. Simulated echoes were then generated by setting the input parameters of the simulator based on the experimental setups. Here, we demonstrate a comparison example in Fig 4. Specifically, we plot an experimental echo signal recorded from a maple tree in Fig 4(a) and plot the corresponding simulated echo signal in Fig 4(b). To compare the statistical properties, we plot the empirical cumulative distribution functions (CDFs) of the signal amplitudes and compared them in Fig 4(c). A visual inspection of Fig 4(a) and 4(b) suggests that the measured and simulated echoes are qualitatively comparable. From the empirical CDFs, we see that the amplitude distributions of the measured and simulated echoes are close to each other, with the simulated amplitudes having slightly heavier tails. More statistical comparisons have been performed after segmenting the echo signals to a few time windows; results were described in [46].

## Results

**Fig 5:**
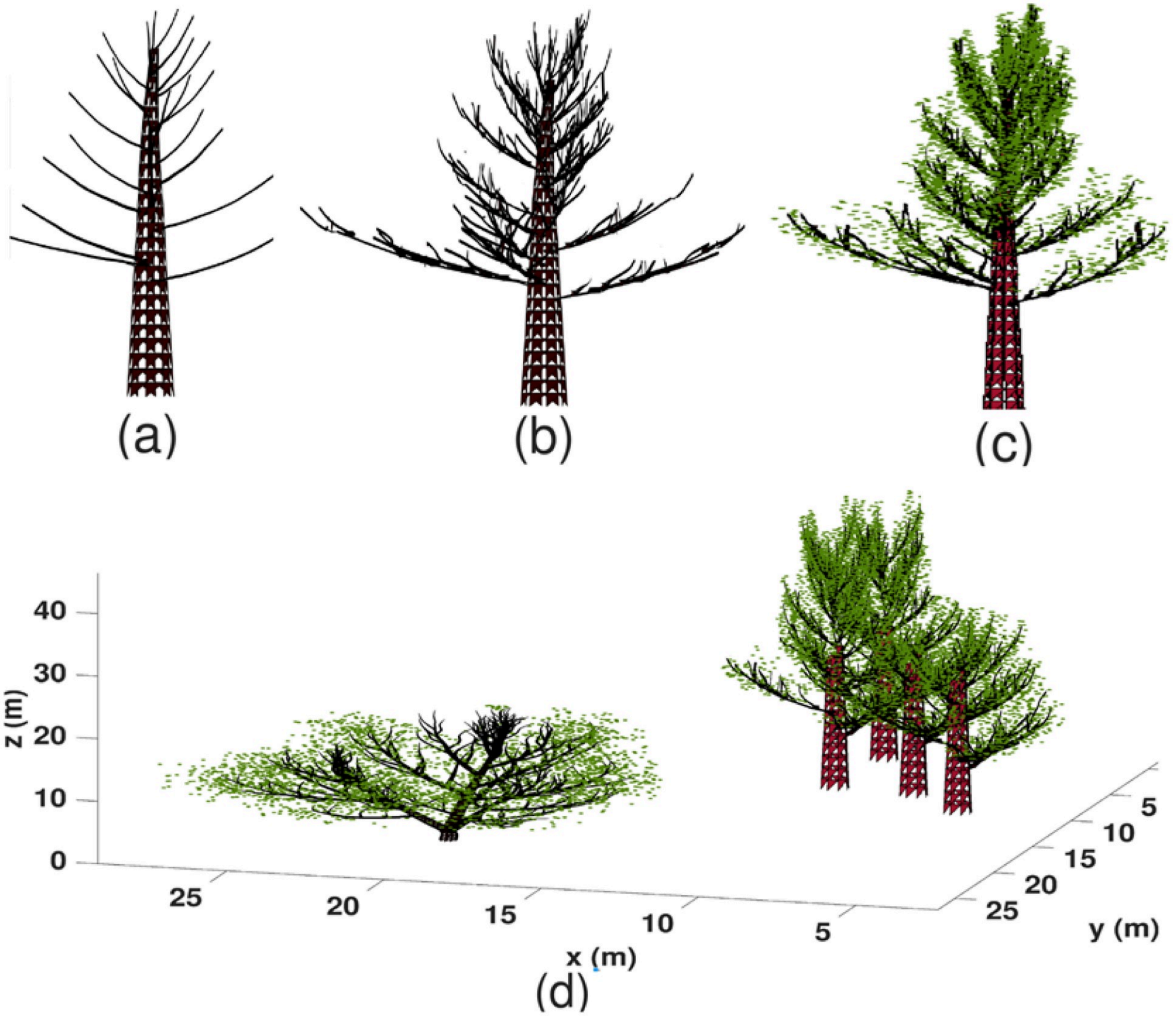
The simulation results of a single tree and a forest. (a) The first level branches generated by the L-system. (b) Sub-branches are added. (c) A view of the complete tree with leaves plotted as green dots. (d) The 3D visualization of a forest with two species of trees.

In this section, we present results of tree simulation and demonstrate outputs of the sonar simulator under designed sensing scenarios. We will also report the computation time by varying different factors under simple designs. All computations were performed in the MATLAB environment by using an Intel^®^ 2 × 16 Core™ E5-2683v4 Broadwell processor with 2.1 GHz frequency and 128 GB, 2400 MHz RAM under Ubuntu 16.04 LTS. In Fig 5(a)–5(c), we show 2-D plots of a Maple tree at different simulation stages. In particular, Fig 5(a) shows the first level branches generated by using the L-system. The branch lengths and angles are adjusted by adopting the geometry of 3D developed CAD models. Fig 5(b) shows the structure after adding sub-branches. Fig 5(c) shows the complete tree with leaves plotted as green dots. Note that in Fig 5(c), while each leaf is simulated as a triangular mesh, only the mid point of these meshes has been plotted as green dots for fast visualization. In Fig 5(d), we demonstrate the 3-D visualization of a simulated forest with five trees. This forest consists of two species—four Maple trees and a Hazelnut tree. Locations of these trees are sampled from IPP in the x-y plane. The intensity function is *λ*_1_(*s*) = 0.4 exp{−(*s*_*x*_ − 5)^2^/5 − (*s*_*y*_ − 5)^2^/6} for Maple trees and is *λ*_2_(*s*) = 0.3 exp{ − (*s*_*x*_ − 15)^2^/3 − (*s*_*y*_ − 15)^2^/6} for the Hazelnut tree.

**Fig 6:**
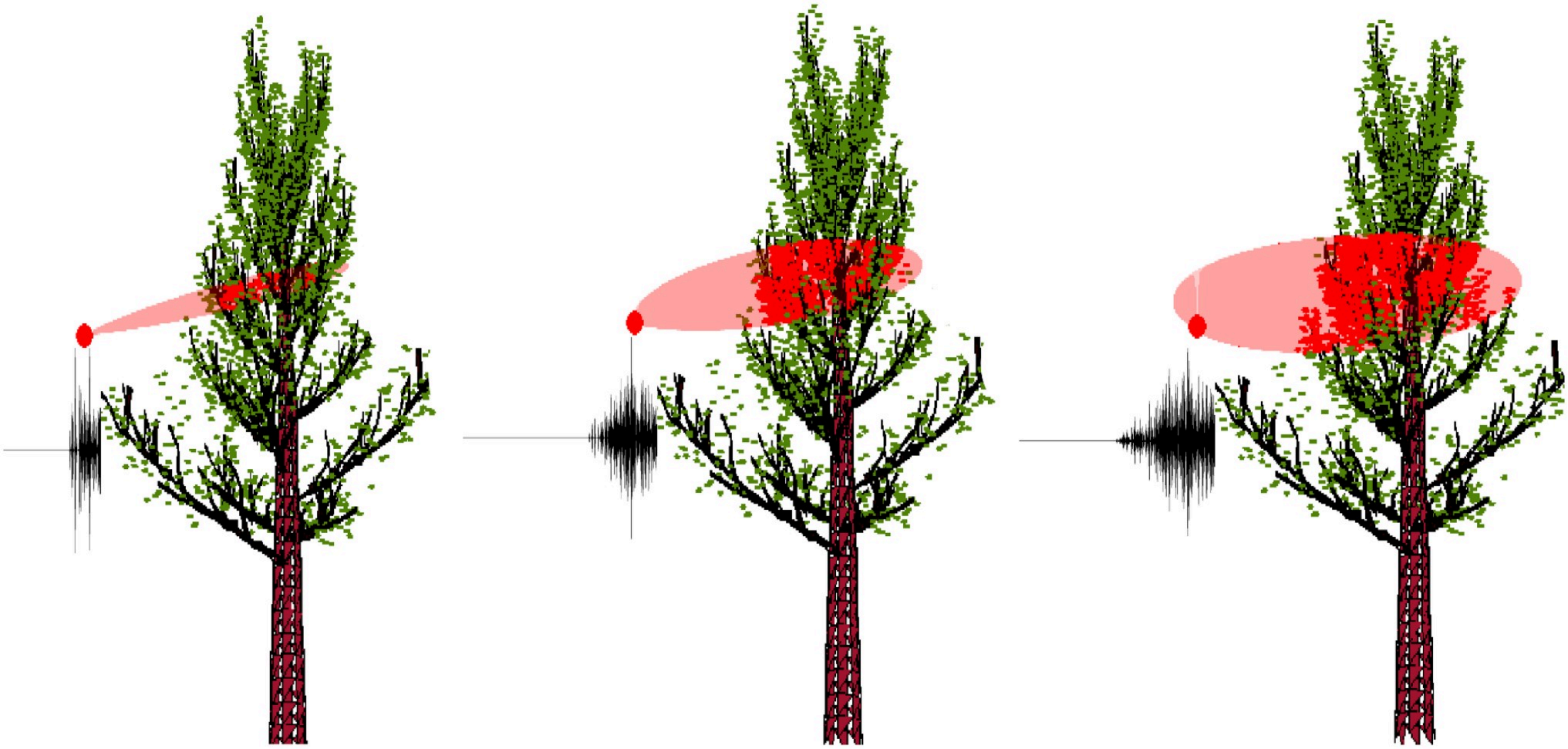
Simulated scenario I. A static sonar with varying beamwidths at 10, 30, 50 degrees (left to right). Red regions highlight the -3 dB contour of the sonar beam. Waveforms of the impulse responses were plotted on the left side of the 2-D view below the sonar location.

To demonstrate performance of the foliage echo simulator in simulated environments, we designed two sensing scenarios—one involves a biosonar in a static mode and the other mimics a UAV in a dynamic motion. In the first scenario, we fixed the position of the sonar while varying its beamwidth. In the second scenario, a UAV was designed to fly a “8” shape route around tree canopies in a simulated forest. The first scenario is shown in Fig 6. The relative position of the sonar and a single tree was fixed. The three figures from left to right demonstrate the effect of varying the -3 dB beamwidth of sonar at 10, 30 and 50 degrees respectively. We used red regions to highlight the -3 dB contour of the sonar beam. Leaves that were located within the -3 dB beam contour were colored in red. Waveforms of the corresponding impulse responses were also plotted along with the scene under each setup. From Fig 6, we see that as the beamwidth increases, more leaves are included in the -3 dB contour of the sonar beam, and the (emission and reception) sonar gain at each scenario also varies. As more leaves reflect sound to the sonar, the time axis of the received waveform is more densely populated, which leads to denser waveforms with more peaks. Furthermore, the values of peaks also appear to be more homogeneous.

**Fig 7:**
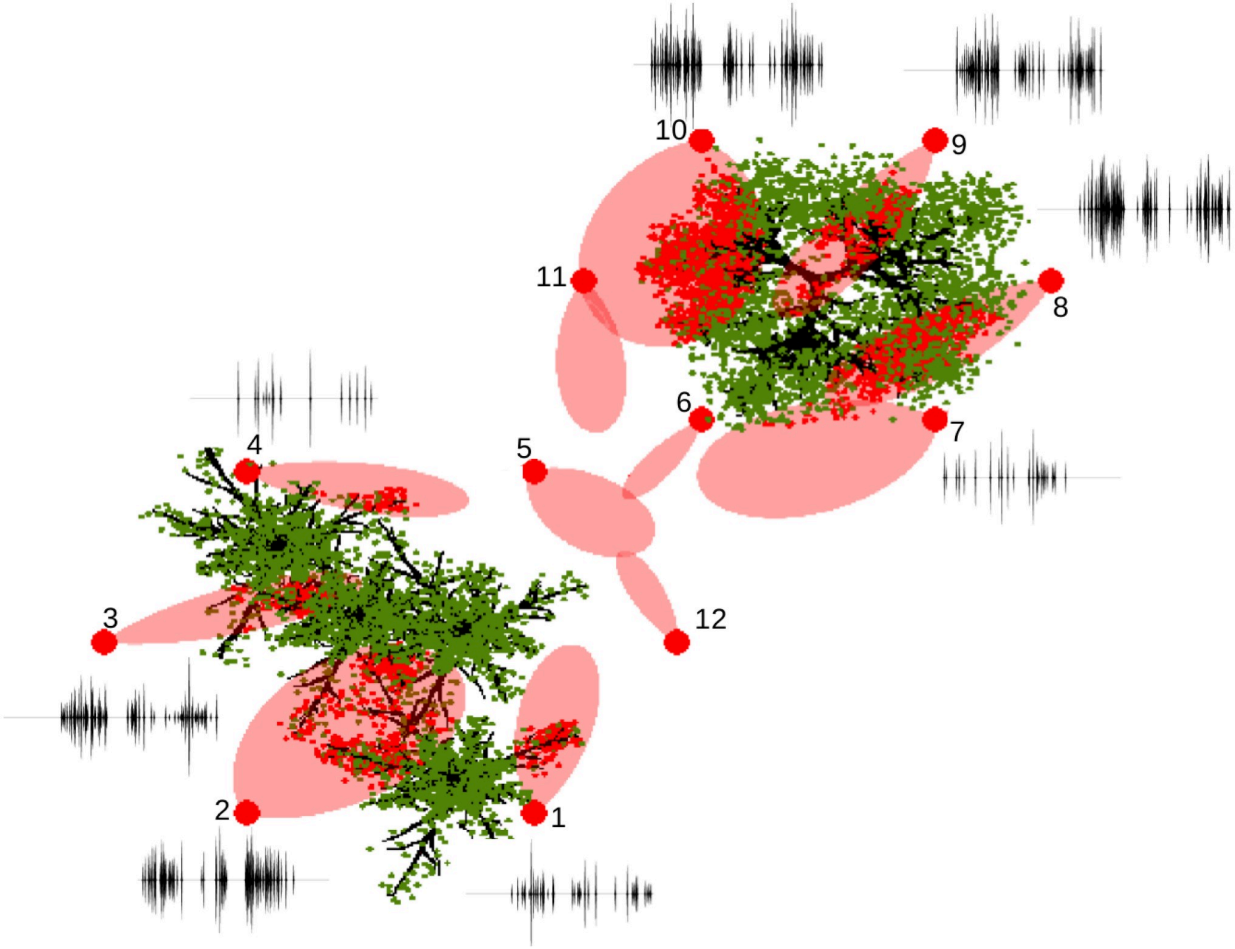
Simulation scenario II. A UAV navigates across a simulated forest following a “8” shaped route. Twelve sampling points are taken along the route. Sonar beamwidth varies. The -3 dB contour of the sonar beams are highlighted in red. The waveforms of impulse responses are plotted at each sampling location.

A view of the second scenario is shown in Fig 7. In this scenario, we simulate a biosonar-based UAV that mimics the flying motion of a bat. In particular, the UAV flies around the canopies of trees in a forest following a “8” shaped route. The simulated forest contains five trees consisting of two species, which is the same scene shown in Fig 5(d). The flying route is produced by using the path-following algorithm of [52]. On the flying route, a total of 12 sampling points were taken. At each sampling point, the direction of the sonar beam was designed to target at the trees, and the beamwidth of the sonar was randomly sampled from the interval of [30, 65] degrees, a range that was found to be common for bat’s biosonar [53]. Fig 7 demonstrates a bird view of the scene along with the -3 dB contours of the sonar beams (highlighted in red) and the resulting impulse responses. Leaves within the sonar beams were plotted as red dots. The sonar beam encounters leaves at seven instances and the corresponding impulse responses are plotted. In the remaining five instances, no leaves are encountered. From Fig 7, we can visualize how spatial distributions of leaves affect the waveform of the impulse responses. Specifically, when there is no leaf in the sonar beam, no signal is received; as more leaves are included in the sonar beam, more peaks are observed in the impulse response. Furthermore, the width and orientation of the sonar beam seem to influence the amplitude of the impulse responses; spatial locations of the leaves in the sonar beam appear to influence the arrival times of the peaks in the impulse response. These observations suggest the possibility of estimating the spatial distribution of leaves from the impulse responses via machine learning models.

Our simulation framework makes it convenient to perform various analyses under different setups. To access the computation complexity of the foliage echo simulator in simulated environments, we designed a simple scene and reported the computation time under different setups. The scene involves a cluster of Maple trees. The number of trees in cluster varies from one to five and the center of cluster was set to be the mean position of the tree locations. We reported the total computation time for calculating impulse responses under two setups. Under the first setup, a sonar-based UAV navigated around the tree canopies following a circular path. We varied the sampling positions of the circular path. At each sampling position, the sonar fired an impulse towards the center of the tree canopies and obtained one impulse response. We report the total computation time for all impulse responses while varying the number of sampling locations and the number of trees in the cluster. For this setup, the sonar beamwidth was fixed at 10 degree. Under the second setup, a sonar-based UAV was set at a fixed location. We reported the computation time for calculating a single impulse response under different sonar beamwidths and different numbers of trees in the cluster.

Results are reported in Table 1. We observe that, the total computation time increases as we increase the number of sampling positions on the flying path or the sonar beamwidth. When we increase the number of trees in the cluster, the leaf densities increase, which also lead to longer computation time. Overall, as all computation time is on the scale of seconds, our foliage echo simulator can perform fairly well in real-time under our simulated framework.

**Table 1:** The computation time under two setups.

Setups	Varying Factors	Computation Time (s)				
	**Number of sampling positions**	**Number of trees**				
		T = 1	T = 2	T = 3	T = 4	T = 5
I. Circular path	1	0.73	0.77	0.86	0.93	1.07
	5	0.88	1.00	1.19	1.33	1.52
	10	0.97	1.21	1.42	1.72	2.11
	15	1.03	1.68	1.92	2.54	3.03
	**Beamwidths in degree**	**Number of trees**				
		T = 1	T = 2	T = 3	T = 4	T = 5
II. Fixed Position	10	0.73	0.77	0.86	0.93	1.07
	20	0.75	0.82	0.89	0.96	1.09
	30	0.79	0.86	0.97	1.06	1.23
	40	0.83	0.91	1.11	1.19	1.42
	50	0.96	1.12	1.33	1.37	1.63

Compared with existing work on biosonar or biosonar-based UAVs, the results presented in this section demonstrate a few unique contributions of the proposed simulation framework. First, while existing studies such as [20, 23], and [21] focus on designing biosonar systems for specific tasks such as landmark tracking, safe landing, and obstacle avoidance, our work focuses on the more fundamental research on providing a general simulation platform. The above results illustrate that this platform can produce random forests that consist of natural-looking trees with multiple species. Together with the foliage echo simulator, this simulation framework can be used to design both static and dynamic sensing scenarios. The resulting simulated data can be used to develop new biosonar-based UAV systems. Second, while existing studies have also proposed ways to simulate foliage echo signals, most of them, such as [16] and [46] have modeled tree leaves as uniformly distributed reflectors and ignored tree structures. Although authors in [47] do consider a tree structure, only a simple scene with one fixed tree is modeled. In contrast to these research works, our proposed framework generates random forests with rich geometric information of trees which is not available in other existing studies.

## Conclusion

We have developed a new computational approach for simulating natural sensing environments and generating biosonar echoes under various sensing scenarios. By integrating L-systems with 3D CAD developed object files, our approach can be used to simulate random, natural-looking trees that contain full geometry about the branches and leaves. These simulated trees can be further combined with a spatial point processes to form a random forest. While we have primarily focused on the simulation of biosonar sensors by using a foliage echo simulator, our approach can be generally used to create 3D virtual environments for the simulation of other types of sensors such as radar and LiDAR.

Along with the proposed foliage echo simulator, our proposed simulation framework provides a convenient platform for the development of smart sonar sensors and bat-inspired UAVs. With this simulation framework, large amount of impulse responses can be simulated under predefined sensing scenarios, based on which, efficient statistical models can be developed and trained to estimate environment parameters that are essential for sensing and navigation.

## Discussion

In our validation study of the foliage echo simulator, simulated and experimental echoes have been compared under a simple setup with a fixed tree and a static sonar. In addition to these results, we believe that performing a larger scale validation study for more complicated environments considered in this paper is a critical next step. Specifically, a validation study can be performed by simulating an sensing environment that is close to a real-world experimental site. This can be done by measuring structures of trees in the experimental site using Lidar and extracting the related geometric information about trees. A prototype—a drone equipped with a biosonar head—can be used to collect experimental echoes by following a designed route under the experimental setup, and data can be compared with simulated data.

In our current study, the trajectories taken by the sonar-based UAV were predefined and we only analyzed the impulse responses generated at different time instances along the trajectories. An interesting future step is to extend it to an active navigation scenario in which an optimal path can be calculated and the sampling position along the path can be determined automatically in real-time. Another potentially interesting step is to extend the framework towards task and motion planning in large knowledge-intensive domains, as recently done by [54] and [55].

To further improve the proposed simulation approach, our future study involves modeling the shading effect between leaves, for example by using an adjusted attenuation function in the foliage echo simulator. Furthermore, a more delicate foliage-echo simulator that takes into account more complicated leaf shapes is also desirable.

Additionally, we have focused on simulating biosonar sensors with a foliage echo simulator. It is worth pointing out that natural environments can have many reflectors other than leaves—tree trunks, branches, rocks, flowing water, etc. The relative importance of these different scatterers in contributing to the echoes will depend on the environment. Considering a bat flying along a forest edge where there are tens of thousands of leaves in front of most of trunks and large branches, considering only the leaves is probably a fair approximation, but it will not always hold for all outdoor scenarios.

Finally, as the primary focus of this paper is on simulating natural sensing environments, our foliage echo simulation model does not take into account nonlinear effects of the UAV’s velocity, i.e., Doppler shifts. Doppler shifts could become significant for fast-flying UAVs that are headed away or towards the foliage. At slow flight speeds or flight paths in parallel with a foliage edges, Doppler effects should not be much of an issue. To simulate scenarios where Doppler shifts become really relevant, the model presented here can be readily extended to include a frequency shift associated with each reflector.

Algorithms developed in this paper have been made available on the Github website [56]. The Github repository can be accessed via the link https://github.com/cfpss/cfpss.github.io.

## Supporting information

S1 File (BST)
